# Impact of secondhand smoke exposure in former smokers on their subsequent risk of coronary heart disease: evidence from the population-based cohort of the Tehran Lipid and Glucose Study

**DOI:** 10.4178/epih.e2020009

**Published:** 2020-03-08

**Authors:** Masoumeh Sadeghi, Maryam S. Daneshpour, Soheila Khodakarim, Amir Abbas Momenan, Mahdi Akbarzadeh, Hamid Soori

**Affiliations:** 1 Department of Epidemiology, School of Public Health and Safety, Shahid Beheshti University of Medical Sciences, Tehran, Iran; 2 Cellular and Molecular Endocrine Research Center, Research Institute for Endocrine Sciences, Shahid Beheshti University of Medical Sciences, Tehran, Iran; 3 Prevention of Metabolic Disorders Research Center, Research Institute for Endocrine Sciences, Shahid Beheshti University of Medical Sciences, Tehran, Iran; 4 Safety Promotion and Injury Prevention Research Center, Shahid Beheshti University of Medical Sciences, Tehran, Iran

**Keywords:** Smoking, Coronary heart disease, Cohort studies, Tehran Lipid and Glucose Study

## Abstract

**OBJECTIVES:**

Cigarette smoking is an established, strong, and modifiable risk factor for coronary heart disease (CHD). However, little research has investigated CHD risk in former smokers who continue to be exposed to others’ cigarette smoke (former & secondhand smokers).

**METHODS:**

In the Tehran Lipid and Glucose Study, a prospective population-based cohort (n=20,069) was followed up for a median period of 14.6 years. A subset of 8,050 participants of 30 years of age and older was analyzed, with first CHD events as the study outcome. Participants were categorized as never, former, current, secondhand, and former & secondhand smokers. Data on smoking intensity (cigarette/d) were also collected. A Cox proportional hazards regression model was applied to estimate the risk of CHD, taking into account the main potential confounders.

**RESULTS:**

The mean age of participants was 46.10 ±11.38 years, and they experienced 1,118 first CHD events (with most CHD cases in former smokers) during the follow-up period. The risk of CHD was highest in current smokers, followed in order by former & secondhand, former, and secondhand smokers (hazard ratio [HR], 1.99; 95% confidence interval [CI], 1.65 to 2.39; HR, 1.55; 95% CI, 1.15 to 2.08; HR, 1.39; 95% CI, 1.12 to 1.72; HR, 1.27; 95% CI, 1.07 to 1.51, respectively), compared to never smokers. The risk of CHD increased with smoking intensity, which has been proposed as a preferable measure of smoking, indicating a dose-response pattern.

**CONCLUSIONS:**

The elevated risk of CHD in former & secondhand smokers was a noteworthy finding, with possible implications for health policy; however, further research is needed.

## INTRODUCTION

Despite remarkable advances in the treatment of cardiovascular disease (CVD)—and in particular, coronary heart disease (CHD)—CVD and its clinical sequelae continue to be the leading cause of morbidity and mortality worldwide [1-3]. Based on the available evidence, CHD is a major concern for global health, as well as a major barrier to achieving the Sustainable Development Goals [1,4]. CHD has been found to be the main cause of death in Iran, and it is projected that the years of life lost due to CHD will increase dramatically in the coming years [5,6].

Overall, 1.4 billion adults worldwide smoke, of whom 1.12 billion are males and 279 million are females [7]. Although the prevalence of current smokers has decreased over time in several countries, the global absolute number of smokers has increased owing to population growth [8]. Cigarette smoking is an established, strong, and modifiable risk factor for CHD [9-11]. In recent years, several observational studies have focused on the associations between cigarette smoking and CVD, myocardial infarction (MI), CHD, and stroke [12-15]. Some studies have addressed smoking behavior as a simple dichotomous (smoker/non-smoker) variable, while a few prospective cohort studies have operationalized cigarette smoking in multiple ways, such as smoking status (never, former, and current smoker), pack/yr, duration, and age of onset of smoking in various age groups and communities [16,17]. Based on the Framingham cohort study findings, former heavy smokers have significantly elevated CVD risk extending beyond 5 years after cessation compared to never smokers [18]. A recently published systematic review and meta-analysis indicated that smoking only about 1 cigarette/d resulted in an additional risk of developing CHD and stroke that was much greater than expected [9].

Although our knowledge base in relation to the effect of cigarette smoking (current and former vs. never smokers) on CHD risk has expanded over the last decade, comparatively little research has been conducted on secondhand smokers and former smokers who are still exposed to others’ cigarette smoke. The limitations of published data leave open important questions about the magnitude and effect size of CHD risk in individuals who are exposed to others’ cigarette smoke after cigarette smoking quitting/cessation.

In an attempt to fill this evidence gap, the present study set out to explore the influence of smoking status (former, current, and secondhand smoking, and in particular secondhand exposure following smoking cessation compared to never smoking) as well as smoking intensity (as another proposed measure of cigarette smoking) on the subsequent risk of CHD.

## MATERIALS AND METHODS

### Study design

The Tehran Lipid and Glucose Study (TLGS) is an open-ended prospective population-based cohort study of a representative sample of residents of Tehran (the capital city of Iran) who were 3 years of age and older at the time of recruitment. This study originated in March 1999 to December 2001, and follow-up and data collection were planned to be accomplished at 3-year intervals. Briefly, 15,005 individuals participated in the first examination and 3,550 individuals were added in the second examination. Newborn children of families were added to the study population after they reached 3 years of age during the follow-up. The study population includes 20,069 individuals, and to date, the median follow-up time of this cohort is 14.66 years (interquartile range [IQR], 10.45 to 6.22). The details of the TLGS cohort study have been described elsewhere [19-21].

### Study sample

**Figure 1.**
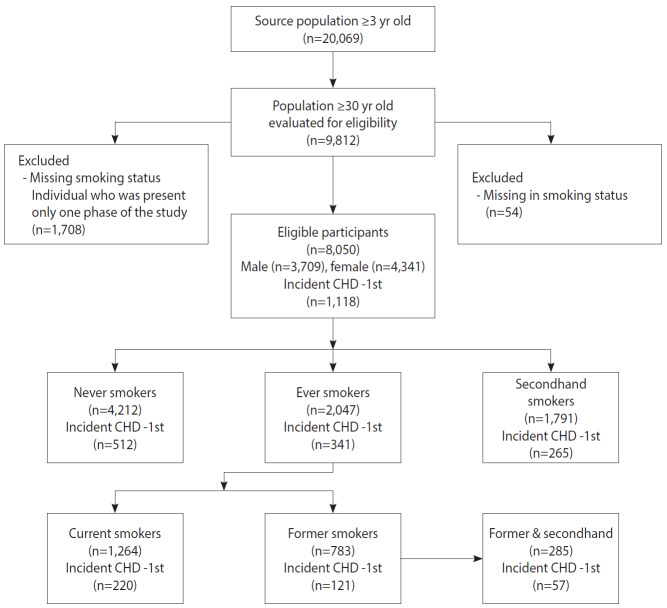
Flow diagram of study design and participants in the Tehran Lipid and Glucose Study cohort (1999-2018). CHD, coronary heart disease.

A subset of 8,050 participants from the TLGS cohort was considered. The included individuals were restricted to those who were 30 years of age and older. Furthermore, participants who had a positive history of CVD (prevalent cases) at the baseline examination, had missing values for either CHD or smoking status, or participated in only 1 phase of the study were excluded (Figure 1).

### Exposure: cigarette smoking status

Participants’ cigarette smoking status was determined based on self-reported smoking behavior. Collected data on participants’ history of current and previous smoking were used to categorize individuals as never, secondhand, former, former & secondhand, and current smokers. Participants were considered to be ever smokers if they had smoked more than 100 cigarettes in their lifetime, while those who had not smoked cigarettes at all or had smoked 100 or fewer cigarettes in their lifetime and did not currently smoke were classified as never smokers. Ever smokers were assigned into 2 groups: participants who had smoked more than 100 cigarettes in their lifetime and had smoked in the last 28 days were considered to be current smokers, while ever smokers who had smoked more than 100 cigarettes in their lifetime but had quit smoking were considered as former smokers. Secondhand smokers (or those exposed to environmental tobacco smoke) were defined as never smokers who were exposed to cigarette smoke at home or at work. Former smokers who were exposed to others’ cigarette smoke were defined as former & secondhand smokers. Information was also gathered on age at initiation of cigarette smoking (categorized as ≤ 17 years old and > 17 years old), smoking intensity (number of cigarettes used per day), and history of smoking cessation (years).

### Outcome assessment

All participants of the TLGS are followed up for any medical events during the preceding year by a telephone call, and are asked about any medical conditions by a trained nurse. In the present study, the outcome of interest was first CHD events. In order to gather outcome data, all participants were followed up annually for fatal or non-fatal CHD (definite MI, probable MI, unstable angina pectoris, angiography-proven CHD and unstable angina pectoris, angiography-proven CHD, and CHD death, which are comparable to the categories in the International Classification of Diseases, 10th revision). CHD events leading to hospitalization or death were confirmed by an outcome committee (cohort outcome panel) consisting of the principal investigator, an internist, an endocrinologist, a cardiologist, an epidemiologist, and a physician (general practitioner) who collected outcome data [22,23].

### Covariates

The potential covariates assessed in the present study were age, sex, body mass index (BMI), high-density lipoprotein (HDL) cholesterol (mg/dL), low-density lipoprotein (LDL) cholesterol (mg/dL), history of type 2 diabetes mellitus (T2DM), systolic blood pressure (SBP, mmHg), diastolic blood pressure (DBP, mmHg), and educational level (less than high school, high school and diploma, more than high school [college education]). Detailed information on anthropometric, clinical, and laboratory measurements have been described elsewhere [22,24].

### Statistical analysis

A Cox proportional hazard regression model was used to estimate the hazard ratios (HRs) and 95% confidence intervals (CIs) for CHD incidence after statistically and graphically assessing and confirming the proportional hazard assumption. A multivariable Cox model was used to estimate the HRs of CHD risk adjusted for age, sex, education level, and the aforementioned potential confounders stratified by smoking status (never, former, current, secondhand, and former & secondhand). HRs were estimated in 4 models with increasing numbers of covariates, as follows: model 1, smoking status alone; model 2, age as a covariate; model 3, age and sex as covariates; and model 4, model 3 plus education level, BMI, history of T2DM, SBP, DBP, HDL cholesterol, and LDL cholesterol.

Since smoking intensity (cigarette/d) has been proposed as a preferable measure of smoking behavior for modeling cardiovascular outcomes [17], we estimated crude and fully adjusted HRs for CHD risk stratified by smoking intensity (≤ 10, 10-20, and ≥ 20 cigarette/d). All statistical analyses were carried out using Stata version 14.0 (StataCorp., College Station, TX, USA) and SPSS 16.0 (SPSS Inc., Chicago, IL, USA). All statistical tests were 2-tailed with significance level of α< 0.05. There were relatively few missing values (minimum for sex [0.2%] and maximum for education level [5.1%]) for all variables.

### Ethics statement

The study was approved by the Shahid Beheshti University of Medical Sciences Ethics Committee (No. IR.SBMU.PHNS.REC. 1396.144). Written informed consent was obtained from all participants.

## RESULTS

The eligible participants for this study were 8,050 individuals aged 30 years or older. More of them were females (54.0%) than males, with a mean age of 46.10±11.38 years. During a median follow-up of 14.66 years, the participants experienced 1,118 first CHD events. First CHD events occurred more frequently in males than in females (8.2% vs. 5.6%). The majority (61.0%) of the CHD cases occurred before the age of 65 years. Fewer than 15% of the study population had a history of T2DM and both sexes were, on average, overweight (mean BMI, 27.5±4.6 kg/m^2^). One-quarter of the participants reported a history of cigarette smoking (25.4%), of which 3.4% were females. Among those who reported no history of cigarette smoking, 22.2% were exposed to secondhand smoke, with a higher proportion among females than among males. A considerable proportion of ever smokers (36.2%) had initiated cigarette smoking in adolescence. The baseline participant characteristics are shown in Table 1.

The distribution of the first CHD events and all study covariates stratified by cigarette smoking status is presented in Table 2. Former smokers accounted for the highest proportion of CHD cases, followed by former & secondhand, current, never, and secondhand smokers (24.3%, 20.0%, 17.4%, 12.2%, and 11.6%, respectively). Only 9.4% of current smokers reported that they smoked ≥ 20 cigarette/d, and approximately 70% of the former & secondhand smokers had quit smoking more than 5 years ago.

**Figure 2.**
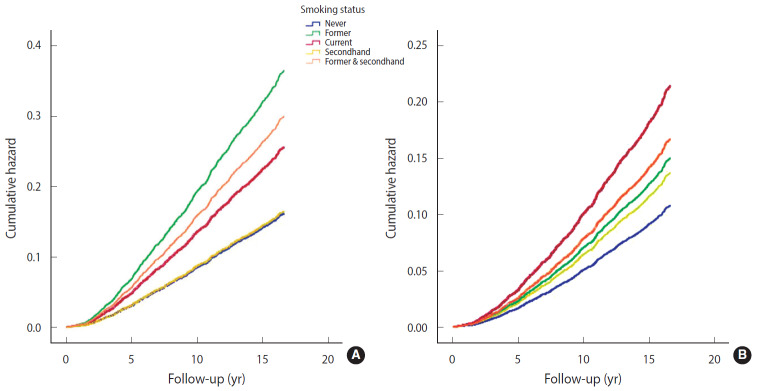
Risk of coronary heart disease based on crude (A) and multivariable fully adjusted model (B) by cigarette smoking status.

In both the crude and multivariable-adjusted models, exposure to cigarette smoking—regardless of being active or passive—was associated with a significantly increased risk of CHD compared to never smoking (Table 3). Surprisingly, the association between cigarette smoking and CHD was stronger in former smokers (HR, 2.26; 95% CI, 1.85 to 2.75) than in current smokers (HR, 1.58; 95% CI, 1.35 to 1.86) in the crude model (Table 3 and Figure 2A). A remarkable finding of the present study was the important and significant risk of CHD in former & secondhand smokers (HR, 1.86; 95% CI, 1.41 to 2.44). After adjusting for age as the main covariate, the associations in former smokers and current smokers changed meaningfully; the risk of CHD increased in current smokers (HR, 2.07; 95% CI, 1.76 to 2.43), but decreased in former smokers (HR, 1.56; 95% CI, 1.28 to 1.91).

It is interesting to note that in former & secondhand smokers, the considerable observed association remained almost unchanged upon adjustment (HR, 1.82; 95% CI, 1.38 to 2.39). In other words, in individuals who were still exposed to others’ cigarette smoke despite quitting smoking, the risk of CHD was 1.8 times more than in never smokers. After controlling for age, sex, education level, BMI, history of T2DM, SBP, DBP, HDL cholesterol, and LDL cholesterol, the risk of CHD was highest in current smokers, followed in order by former & secondhand smokers, former smokers, and secondhand smokers (HR, 1.99; 95% CI, 1.65 to 2.39; HR, 1.55; 95% CI, 1.15 to 2.08; HR, 1.39, 95% CI, 1.12 to 1.72; HR, 1.27; 95% CI, 1.07 to 1.51), compared to never smokers (Table 3, Figure 2B).

As illustrated in the fully adjusted model shown in Table 3, the risk of CHD in males was 1.57 times higher than in females (HR, 1.57; 95% CI, 1.35 to 1.84). Although education level did not have a significant effect on CHD incidence, it did seem that more than 12 years of formal education (more than high school) had a statistically borderline protective effect against CHD, reducing its incidence by nearly 20% (HR, 0.78; 95% CI, 0.59 to 1.01).

When smoking status (the study exposure) was replaced with smoking intensity as another measure of cigarette smoking, evidence of a significant dose-response pattern between a higher number of cigarettes per day and risk of CHD was observed. To summarize, in both the crude and fully adjusted models, the risk of CHD increased with greater smoking intensity (< 10 cigarette/d: HR, 1.65; 95% CI, 1.32 to 2.06; 10-19 cigarette/d: HR, 2.22; 95% CI, 1.75 to 2.80; and ≥ 20 cigarette/d: HR, 2.38; 95% CI, 1.58 to 3.58) (Table 4).

## DISCUSSION

In this large population-based prospective cohort from Iran with a remarkable median length of follow-up, the risk for incident CHD was higher in current, former & secondhand, former, and secondhand smokers (with fully adjusted HRs of 1.99, 1.55, 1.39, and 1.27, respectively) than in never smokers, independently of other CHD risk factors. It is worth emphasizing that the risk of CHD events in participants who had quit smoking but were exposed to others’ cigarette smoke at home or work was 1.55 times higher than that of never smokers, while the risk was 1.39 times higher in those with a history of cigarette smoking (former smokers) and 1.27 times in never smokers who were exposed to others’ cigarette smoke (secondhand smokers). In another study that dealt with the influence of smoking intensity (cigarette/d) on incidence of CHD, a considerable risk of CHD was found to be associated with even light cigarette smoking (1-10 cigarette/d). CHD events occurred 65% more frequently in light smokers than in those with no history of cigarette smoking. This window of hazard for incident CHD was greater for those who smoked more than 10 cigarette/d (more than twice than never smokers). This is a key message for smokers who suppose that light smoking is less risky or harmless.

Our results are consistent with previously conducted large cohort studies from various nations [17,18,25-30] and offer more evidence to support the association between smoking and risk of CHD. In the Framingham heart cohort study, which contained 8,770 participants with a mean age of 42.2±11.8 years, the risk of CVD was meaningfully higher (75%) in current smokers than in never smokers, whereas former smokers—regardless of intensity—were not at an increased risk [18], suggesting that risk may drop as time passes after quitting smoking. The relative risk of CVD events for never smokers exposed to secondhand smoke (secondhand smokers) in comparison with those unexposed (never smokers) was 1.23 (95% CI, 1.16 to 1.31) in a systematic review conducted of 38 large observational studies [31]. Of note, however, the observed association between secondhand smoke exposure and CVD was distinctly stronger among Chinese than among Americans [31].

It is widely supposed that quitting smoking is associated with a significant reduction in the risk of death among patients with CHD. Irrespective of age, sex, index cardiac event, country, and the year of study initiation, this mortality risk reduction seems to be consistent [32].

It is even believed that the risk of sudden cardiac death in smokers who have quit smoking more than 20 years ago is equivalent to that of never smokers [33]. However, those with a history of cigarette smoking are less sensitive to others’ cigarette smoke (secondhand smoke). Studies conducted on environmental tobacco smoke/secondhand smoke suggest that its effects on the risk of CHD are stronger than would be expected based on associations in current smokers who are exposed to higher doses of cigarette smoke [34]. There are some who argue that current smokers may have adaptive responses that lead to lower increases in CHD risk at high levels of exposure compared to the increases in risks experienced by light secondhand smokers [35,36]. The available evidence suggests that cardiovascular system, platelet, and endothelial function [37-39]; atherosclerosis and arterial stiffness [40,41]; oxidative stress [42]; inflammation [43,44]; and infarct size are highly sensitive to the toxins in secondhand smoke. The effects of even short-term exposure (minutes to hours) to secondhand smoke are nearly as large (on average 80-90%) as current smoking [45].

The current analysis confirmed the findings discussed above and provided further evidence for the association between secondhand smoke exposure and the risk of CHD events. In addition, a notable finding of our study was that the risk of CHD in former smokers who were exposed to others’ cigarette smoke (former & secondhand smokers) was higher than in secondhand smokers and former smokers, respectively, and approached that of current smokers. However this group—as a separate category of smoking status—has not been examined in other studies. Since this is an important issue, further research should be done to confirm this finding.

Regarding smoking intensity, our findings also confirmed the results of research conducted in recent years [17,27-29,46,47]. The average baseline smoking intensity (cigarette/d) was significantly associated with incidence of CHD in a dose-response pattern. Slight inconsistencies in the magnitude of the risk among studies may be attributed to diversity in study participants, or may be due to residual confounding and/or interaction with other baseline factors including physical activity, nutritional factors, and genetic factors [48].

Strengths of the present study include the analysis of a large population-based prospective cohort with a considerable sample size and more than 14 years of follow-up. The tracking and frequent follow-up of participants, as well as the in-person assessment of their smoking histories, resulted in a comprehensive picture of exposure. Because a comprehensive range of potential covariates related to CHD was considered, the estimated HRs can be considered valid and accurate. Modeling of smoking history in the context of CHD was performed using 2 distinct measures of cigarette smoking (smoking status and smoking intensity).

Nonetheless, there are several sources of uncertainty in the present study. Although information on participants’ smoking histories was obtained in person, the recorded data on duration of smoking cessation and history of other types of tobacco consumption were not very reliable. Furthermore, recall bias may have influenced the self-reported data on smoking initiation and the exact date of quitting smoking. Our analysis might have underestimated the true HRs because of the social stigma towards cigarette smoking among Iranian females, which may have led to underreporting of cigarette smoking by female participants. Finally, the possibility of residual confounding in our model should be considered, despite careful adjustment for the main confounders.

The findings of this large population-based prospective cohort study indicate that current, former & secondhand, former, and secondhand smokers had an elevated risk of incident CHD (in descending order) compared to never smokers. Furthermore, evidence of a significant dose-response pattern between the number of cigarettes smoked per day (smoking intensity) and the risk of CHD was observed. The finding of a considerably elevated risk of CHD in individuals who were exposed to others’ cigarette smoke after smoking cessation is remarkable. This study has raised this important issue, which needs further exploration. Efforts should be made to convey this message to health policy-makers and government officials to improve effective tobacco control and anti-smoking strategies, especially in communities where the prevalence of smoking is increasing, but laws making public environments smoke-free have not been passed due to a lack of political commitment, provided that our findings are confirmed by future studies.

## Figures and Tables

**Table 1.** Distribution of baseline characteristics of the cohort (TLGS; 1999-2018)

Characteristics	Male (n=3,709)	Female (n=4,341)	Total (n=8,050)
Age (yr)	47.76±12.82	46.10±11.38	46.89±12.11
Incident CHD (first event)	663 (8.2)	455 (5.6)	1,118 (13.8)
Education level (yr)			
Did not graduate	326 (4.3)	375 (4.9)	701 (9.2)
Less than high school (≤ 9)	1,378 (18.1)	2,100 (27.5)	3,478 (45.6)
High school (10-12)	1,156 (15.2)	1,170 (15.3)	2,326 (30.5)
More than high school (>12)	660 (8.7)	467 (6.1)	1,127 (14.8)
History of cigarette smoking			
No history	1,933 (24.1)	4,057 (50.5)	6,003 (74.6)
Positive history	1,770 (22.0)	275 (3.4)	2,047 (25.4)
Smoking status			
Never	1,263 (15.7)	2,949 (36.6)	4,212 (52.3)
Former	674 (8.4)	109 (1.4)	783 (9.7)
Current	1,096 (13.6)	168 (2.1)	1,264 (15.7)
Secondhand	670 (8.3)	1,121 (13.9)	1,791 (22.2)
Age at smoking initiation (yr)	20.65±10.23	26.07±13.53	21.50±10.98
≤17	599 (33.6)	47 (2.6)	647 (36.2)
>17	952 (53.4)	185 (10.4)	1,138 (63.8)
History of using non-cigarette forms of tobacco			
No history	2,699 (33.8)	3,987 (49.7)	6,700 (83.5)
Positive history	983 (12.3)	336 (4.2)	1,320 (16.5)
History of type 2 diabetes			
No diabetes	2,376 (26.5)	2,622 (36.7)	4,229 (63.2)
Pre-diabetes	830 (9.2)	974 (13.6)	1,804 (22.8)
Diabetes	476 (5.1)	633 (8.9)	1,109 (14.0)
No. of cigarette per day (n)	10 [5-20]	12 [5-20]	8 [5-11]
Smoking cessation (yr)	11 [5-20]	11 [4-20]	13 [5-22]
SBP (mmHg)	121.26±18.61	121.05±20.29	121.14±19.53
DBP (mmHg)	78.32 ± 11.04	78.65±10.91	78.50±10.97
LDL-C (mg/dL)	132.05±36.17	139.57±39.89	136.08±38.40
HDL-C (mg/dL)	37.84±9.29	44.60±11.16	41.47±10.87
Non-HDLC (mg/dL)	173.11±41.92	177.61±47.71	175.65±45.35
Body mass index (kg/m^2^)	26.29±3.96	28.53±4.79	27.49±4.56

Values are presented as mean±standard deviation or number (%) or median [range]. TGLS, Tehran Lipid and Glucose Study; CHD, coronary heart disease; SBP, systolic blood pressure; DBP, diastolic blood pressure; LDL-C, low-density lipoprotein cholesterol; HDL-C, high-density lipoprotein cholesterol.

**Table 2.** Description of baseline cohort characteristics stratified by cigarette smoking status (TLGS; 1999-2018)

Characteristics	Smoking status				
	Never (n=4,212)	Former (n=498)	Current (n=1,264)	Secondhand (n=1,791)	Former & secondhand (n=285)
Age (yr)	47.49±12.37	54.25±12.84	44.85±10.76	44.66±11.05	48.32±12.77
Coronary heart disease	512 (12.2)	121 (24.3)	220 (17.4)	208 (11.6)	57 (20.0)
Sex/male	1,263 (30.1)	420 (84.3)	1,096 (86.8)	670 (37.5)	254 (89.1)
Education level (yr)					
Did not graduate	354 (8.8)	50 (10.5)	109 (9.1)	164 (9.8)	26 (9.5)
Less than high school (≤9)	1,969 (49.0)	224 (47.3)	454 (37.8)	727 (43.4)	108 (39.6)
High school (10-12)	1,146 (28.5)	121 (25.5)	454 (37.8)	513 (30.6)	95 (34.8)
More than high school (>12)	549 (13.7)	79 (16.7)	184 (15.3)	271 (16.2)	44 (16.1)
Age at starting smoking (yr)					
≤17	NA	102 (26.4)	502 (40.5)	NA	47 (27.3)
>17	NA	276 (73.5)	737 (59.5)	NA	125 (72.7)
Intensity (cigarette/d)					
<10	NA	77 (88.5)	576 (46.5)	NA	44 (86.3)
10-19	NA	9 (10.3)	548 (44.2)	NA	6 (11.8)
≥20	NA	1 (1.1)	116 (9.4)	NA	1 (2.0)
History of smoking cessation (yr)					
<5	NA	105 (21.4)	NA	NA	83 (29.6)
5-9	NA	90 (18.4)	NA	NA	52 (18.6)
10-14	NA	77 (15.7)	NA	NA	45 (16.1)
15-24	NA	129 (26.3)	NA	NA	55 (19.6)
≥25	NA	89 (18.2)	NA	NA	45 (16.1)
Body mass index (kg/m^2^)					
<18.5	31 (0.7)	4 (0.8)	40 (3.2)	12 (0.7)	5 (1.8)
18.5-24.9	1,056 (25.2)	157 (31.6)	491 (39.0)	455 (25.5)	92 (32.3)
25-29.9	1,844 (43.9)	230 (46.3)	533 (42.3)	792 (44.4)	133 (46.7)
≥30.0	1,267 (30.2)	106 (21.3)	196 (15.6)	524 (29.4)	55 (19.3)
History of type 2 diabetes					
No diabetes	2,514 (61.4)	258 (53.3)	877 (71.8)	1,145 (66.5)	169 (61.0)
Pre-diabetes	987 (24.1)	134 (27.7)	118 (9.7)	222 (12.7)	47 (17.0)
Diabetes	591 (14.4)	92 (19.0)	226 (18.5)	381 (21.8)	61 (22.0)
Blood pressure (mmHg)					
SBP	122.72±20.01	126.20±20.20	115.31±17.13	119.87±18.97	119.86±18.97
DBP	79.47±10.96	79.22±10.99	74.94 ± 10.38	78.51±10.63	78.51±10.63
LDL-C (mg/dL)	43.09±10.92	39.43±9.56	37.37±9.69	41.63±11.17	132.66±36.18
HDL-C (mg/dL)	138.81±39.59	140.28±37.99	131.07±36.31	132.66 ± 36.18	41.63 ± 11.17

Values are presented as mean±standard deviation or number (%). TGLS, Tehran Lipid and Glucose Study; SBP, systolic blood pressure; DBP, diastolic blood pressure; LDL-C, low-density lipoprotein cholesterol; HDL-C, high-density lipoprotein cholesterol; NA, not applicable.

**Table 3.** Multivariable-adjusted risk of CHD stratified by cigarette smoking status (TLGS; 1999-2018)

Variables	No. of people (n)	No. of CHD events (n)	Model 1	Model 2	Model 3	Model 4
Smoking status						
Never	4,212	512	1.00 (reference)	1.00 (reference)	1.00 (reference)	1.00 (reference)
Former	498	121	2.26 (1.85, 2.75)	1.56 (1.28, 1.91)	1.37 (1.11, 1.69)	1.39 (1.12, 1.72)
Current	1,264	220	1.58 (1.35, 1.86)	2.07 (1.76, 2.43)	1.76 (1.48, 2.09)	1.99 (1.65, 2.39)
Secondhand	1,791	208	1.02 (0.87, 1.20)	1.27 (1.07, 1.49)	1.24 (1.05, 1.46)	1.27 (1.07, 1.51)
Former & secondhand	285	57	1.86 (1.41, 2.44)	1.82 (1.38, 2.39)	1.58 (1.19, 2.09)	1.55 (1.15, 2.08)
Age, mean (yr)	-	-	-	1.07 (1.06, 1.07)	1.06 (1.06, 1.07)	1.05 (1.05, 1.06)
Sex						
Female	4,341	455	-	-	1.00 (reference)	1.00 (reference)
Male	3,709	663	-	-	1.37 (1.19, 1.57)	1.57 (1.35, 1.84)
Education (yr)						
Did not graduate	326	113	-	-	-	1.00 (reference)
≤9	1,378	547	-	-	-	0.92 (0.76, 1.15)
10-12	1,156	262				0.88 (0.70, 1.11)
>12	655	134	-	-	-	0.78 (0.59, 1.01)
T2DM						
No diabetes	2,376	468	-	-	-	1.00 (reference)
Pre-diabetes	830	292	-	-	-	1.21 (1.04, 1.42)
Diabetes	476	327	-	-	-	1.91 (1.99, 2.72)
BMI (kg/m^2^)	-	-	-	-	-	1.01 (0.99, 1.01)
SBP (mmHg)	-	-	-	-	-	1.01 (1.00, 1.01)
DBP (mmHg)	-	-	-	-	-	1.01 (1.00, 1.01)
LDL-C (mg/dL)	-	-	-	-	-	1.01 (1.00, 1.01)
HDL-C (mg/dL)	-	-	-	-	-	0.98 (0.98, 0.99)

Values are presented as hazard ratio (95% confidence interval). Model 1: crude model (smoking status alone); Model 2: adjusted for age; Model 3: adjusted for age and sex; Model 4: adjusted for age, sex, education level, T2DM, BMI, SBP, DBP, LDL-C, and HDL-C. CHD, coronary heart disease; TGLS, Tehran Lipid and Glucose Study; T2DM, type 2 diabetes mellitus; BMI, body mass index; SBP, systolic blood pressure; DBP, diastolic blood pressure; LDL-C, low-density lipoprotein cholesterol; HDL-C, high-density lipoprotein cholesterol.

**Table 4.** Multivariable-adjusted risk of CHD stratified by smoking intensity (TLGS; 1999-2018)

Variables	No. of people (n)	No. of CHD events (n)	Model 1	Model 2	Model 3	Model 4
Smoking intensity (cigarette/d)						
Never smoker	6,003	720	1.00 (reference)	1.00 (reference)	1.00 (reference)	1.00 (reference)
<10	830	124	1.36 (1.11, 1.66)	1.71 (1.40, 2.10)	1.52 (1.23, 1.89)	1.65 (1.32, 2.06)
10-19	455	96	1.76 (1.43, 2.15)	2.18 (1.78, 2.68)	1.87 (1.50, 2.33)	2.22 (1.75, 2.80)
≥20	88	24	2.31 (1.58, 2.37)	2.74 (1.87, 3.99)	2.31 (1.56, 3.41)	2.38 (1.58, 3.58)
Age, mean (yr)	-	-		1.07 (1.06, 1.07)	1.07 (1.06, 1.07)	1.06 (1.05, 1.06)
Sex						
Female	4,341	455	-	-	1.00 (reference)	1.00 (reference)
Male	3,709	663	-	-	1.32 (1.14, 1.52)	1.46 (1.24, 1.72)
Education (yr)						
Did not graduate	326	113	-	-	-	1.00 (reference)
≤9	1,378	547	-	-	-	0.88 (0.70, 1.11)
10-12	1,156	262				0.86 (0.67, 1.10)
>12	655	134	-	-	-	0.75 (0.56, 0.99)
T2DM						
No diabetes	2,376	468	-	-	-	1.00 (reference)
Pre-diabetes	830	292	-	-	-	1.25 (1.06, 1.47)
Diabetes	476	327	-	-	-	2.48 (2.10, 2.94)
BMI (kg/m^2^)	-	-	-	-	-	1.02 (1.00, 1.03)
SBP (mmHg)	-	-	-	-	-	1.01 (1.00, 1.01)
DBP (mmHg)	-	-	-	-	-	1.02 (1.00, 1.02)
LDL cholesterol (mg/dL)	-	-	-	-	-	1.01 (1.00, 1.01)
HDL cholesterol (mg/dL)	-	-	-	-	-	0.98 (0.97, 0.99)

Values are presented as hazard ratio (95% confidence interval). Model 1: crude model (smoking status alone); Model 2: adjusted for age; Model 3: adjusted for age and sex; Model 4: adjusted for age, sex, education level, T2DM, BMI, SBP, DBP, LDL cholesterol, and HDL cholesterol. CHD, coronary heart disease; TGLS, Tehran Lipid and Glucose Study; T2DM, type 2 diabetes mellitus; BMI, body mass index; SBP, systolic blood pressure; DBP, diastolic blood pressure; LDL-C, low-density lipoprotein cholesterol; HDL-C, high-density lipoprotein cholesterol.
